# Malocclusions, pathologic tooth migration, and the need for orthodontic treatment in subjects with stage III–IV periodontitis. A cross-sectional study

**DOI:** 10.1093/ejo/cjad003

**Published:** 2023-03-04

**Authors:** Eglė Zasčiurinskienė, Liveta Rastokaitė, Rune Lindsten, Nomeda Basevičienė, Antanas Šidlauskas

**Affiliations:** Department of Orthodontics, Faculty of Odontology, Medical Academy, Lithuanian University of Health Sciences, Kaunas, Lithuania; Faculty of Odontology, Medical Academy, Lithuanian University of Health Sciences, Kaunas, Lithuania; Department of Orthodontics, Institute for Postgraduate Dental Education, Jönköping, Sweden; Centre for Oral Health, School of Health and Welfare, Jönköping University, Sweden; Department of Dental and Oral Pathology, Faculty of Odontology, Medical Academy, Lithuanian University of Health Sciences, Kaunas, Lithuania; Department of Orthodontics, Faculty of Odontology, Medical Academy, Lithuanian University of Health Sciences, Kaunas, Lithuania

## Abstract

**Background:**

Literature is scarce on malocclusion prevalence and orthodontic treatment need (OTN) in subjects with stage III–IV periodontitis. Study aims were to assess prevalence of primary and secondary malocclusions in subjects with stage III–IV periodontitis and OTN based on pathologic tooth migration (PTM) and occlusal trauma of anterior teeth (AT).

**Subjects and methods:**

One hundred and twenty-one subjects with stage III–IV periodontitis were examined. A comprehensive periodontal–orthodontic examination was performed. Exclusion criteria: age <30 years, removable prosthetics, uncontrolled diabetes, pregnancy/lactation, and oncologic disease.

**Results:**

Class II malocclusion was found in 49.6% (Class II div 1—20.7%, Class II div 2—9.9%, subdivision Class II—19.0%), Class I—31.4%, Class III—10.7%, no malocclusion—8.3% of subjects. PTM was observed in 74.4% of maxillary and 60.3% of mandibular AT. Spacing and extrusion were the main types of PTM of AT. Odds ratio for PTM of maxillary AT was 9.3 in cases with >30% of sites with clinical attachment loss ≥5 mm (*P* = 0.001). Spacing of maxillary AT was influenced by periodontitis, Class III malocclusion, and lost teeth. Tongue habit had impact on spacing of mandibular AT. Dental Health Component of Index of Orthodontic Treatment Need yielded that OTN was found in >50% and OTN due PTM, occlusal trauma and impaired function in 66.1% of subjects.

**Conclusions:**

The most prevalent malocclusion was Class II. Spacing and extrusion were prevalent types of PTM of AT. OTN was found in more than half of the subjects. The study highlights the need for preventive measures for PTM in subjects with stage III–IV periodontitis.

## Introduction

Periodontitis is a chronic, plaque-induced, inflammatory disease leading to the deterioration of tooth-supporting tissues (1). The disease results from the imbalance between plaque bacteria and the host immune response (2). Familial anamnesis, systemic health disturbances (e.g. diabetes), behavioural and environmental risk factors such as smoking and diet and decreased immune regulation due to ageing have been described as predispositions to increased susceptibility to the disease (3–8). Local factors, such as ‘hanging’ dental restorations and occlusal trauma, have been found to be associated with deeper probing depths and poorer periodontal prognosis (9, 10). There is an on-going debate in the literature that malocclusion may have a co-destructive influence on the disease progression; however, due to the lack of evidence in human clinical studies, the role of occlusion in periodontal disease is still under question (5, 11, 12).

Periodontal disease has a prevalence up to 50%, but advanced stages of periodontitis have been found to affect from 7.8 to 9.8% of the adult population (13, 14).

A new case definition of periodontitis (staging and grading) was introduced in 2017 based on interdental clinical attachment loss (CAL), alveolar bone loss, number of lost teeth due to periodontitis, and the rate of progression of the disease (15, 16). Periodontitis stages I and II are initial, not exceeding CAL of 5 mm, probing pocket depth (PPD) up to 6 mm and no tooth loss. Periodontitis stages III and IV include the more severe cases of the disease with greater CAL (≥5 mm), greater probing depths (≥6 mm), horizontal and vertical alveolar bone defects and furcation involvement, together with numbers of teeth lost due to periodontitis. Periodontitis grade describes the rate (slow, moderate, and rapid) of progression modified by factors such as cigarette smoking, metabolic control of diabetes, and age (in relation to bone loss) (15, 16). Loss of posterior teeth and decreased height of posterior occlusion, increased numbers of teeth with CAL loss, pathologic migration of anterior teeth, impaired function, and worsened smile aesthetics are the main consequences of the disease (17, 18). Furthermore, impaired chewing function and poor smile aesthetics lead to poor quality of life and impaired physical and psychosocial well-being and are the common reasons for most patients to seek treatment (18–21).

Treatment of subjects with stage III–IV periodontitis and secondary malocclusions is complex, including a team approach (17, 22, 23). Orthodontic treatment (OT) is more often included in the multidisciplinary treatment for adult patients with stage III–IV periodontitis due to impaired smile aesthetics, functional problems, or as an adjunctive treatment prior to prosthetic rehabilitation. Sometimes, orthodontic therapy is the only choice for the conservative rehabilitation of occlusion, function, and aesthetics in subjects with stage III–IV periodontitis (5, 24, 25). A questionnaire study showed that patients do not have enough information about OT possibilities for full occlusal rehabilitation, which can probably be explained by the lack of knowledge in the dental community (26). Interestingly, a large percentage of subjects who had undergone orthodontic consultation was willing to undergo combined periodontal–orthodontic treatment to preserve natural dentition (26).

The effect of orthodontic tooth movement on changes in periodontal tissues in subjects with advanced stages of periodontitis has been studied in numerous clinical studies, where improvement of periodontal parameters, alveolar bone changes, and some risk for external root resorption have been reported (27–30). Alternative treatment options, such as splinting of teeth and occlusal adjustment, have also been described. However, those alternatives are often not suitable for the advanced stages of periodontitis with severe migration of teeth (31). Full mouth prosthetic rehabilitation, usually with multiple extractions of hopeless teeth, tooth or implant-supported prosthesis, has also been described as an alternative to OT. Interestingly, implants, which were often used for substitution of teeth with poor periodontal prognosis, have now discovered to have a high risk of peri-implantitis, especially in subjects with periodontitis (32, 33). Therefore, with its benefits of preservation of natural dentition and its positive effect on tooth surrounding tissues and management of pathologic tooth migration (PTM), OT, even in advanced stages of periodontitis, is becoming increasingly popular both among patients and dentists.

Although the phenomenon of PTM is well described, the literature is scarce on the prevalence of primary and secondary malocclusions in stage III–IV periodontitis and the nature of tooth migration in different sagittal malocclusions. The role of occlusal interferences and oral habits in the aetiology of PTM is also scarcely studied, even though these factors are often observed in subjects with advanced stages of periodontitis (22, 34, 35). The need for OT in stage III–IV periodontitis is unknown.

The aim of the present cross-sectional study was to assess the prevalence of primary and secondary malocclusions in subjects with stage III–IV periodontitis. The secondary aim was to assess OT need based on malocclusions, PTM, and occlusal trauma of anterior teeth.

## Materials and methods

Approval by the Regional Ethical Review Board, Kaunas, Lithuania, was obtained (No. BE-2-111; 2019-12-20). The study followed the ethical principles of declaration of Helsinki and STROBE guidelines for cross-sectional studies (36). Detailed oral and written information about the study’s aim and design was provided before the study. All patients signed informed consent to undergo periodontal–orthodontic examination.

### Subjects

Selection of subjects was limited to the period of August 2020 to January 2022. Individuals with periodontal disease consecutively attending the Department of Dental and Oral Pathology at the Lithuanian University of Health Sciences (LUHS), Kaunas, Lithuania, were invited to participate in a research study and undergo comprehensive periodontal–orthodontic examination. After signing informed consent forms, subjects were selected according to inclusion and exclusion criteria. Inclusion criteria were: 1. age ≥30 years; 2. diagnosis of periodontitis stage III or IV (15). Exclusion criteria were the following: 1. non-inflammatory periodontal disease; 2. removable prosthetic dental appliances; 3. pregnant or lactating women; 4. uncontrolled diabetes; 5. oncologic disease. Subjects with other systemic diseases were not excluded but carefully recorded.

### Periodontal examination

All subjects were clinically examined by an experienced investigator (EZ). The evaluation was performed on the six surfaces around each tooth with a periodontal probe (Hu-Friedy PCP-UNC 15, Chicago, Illinois, USA). The data were recorded in periodontal charts for the study. The following measurements were recorded:

- Visible plaque index: evaluated by the presence of dental plaque on the six sites of each tooth [mesiobuccal (MB), buccal (B), distobuccal (DB), mesiolingual (ML), lingual (L), and distolingual (DL)].
- Bleeding on probing: recorded bleeding sites immediately after probing.
- PPD: measured distance from the gingival margin to the apical portion of the gingival sulcus in millimetres. Data were recorded on all six sites of a tooth (MB, B, DB, ML, L, and DL).
- Gingival recession (REC): measured distance from the cementoenamel junction (CEJ) to the gingival margin in millimetres. Data were recorded on all six sites of the tooth (MB, B, DB, ML, L, and DL).
- CAL: measured as a distance from the apical portion of the gingival sulcus to the CEJ in millimetres. Data were recorded on all six sites of the tooth (MB, B, DB, ML, L, and DL).
- Tooth mobility was assessed by touching the tooth with the index finger on one side and applying a compressive force with an instrument on the other side. Mobility was scored: 1—tooth moves less than 1 mm in the buccolingual or mesiodistal directions, 2—tooth moves 1 mm or more in the buccolingual or mesiodistal directions but does not move in the vertical direction, and 3—tooth moves 1 mm or more in buccolingual or mesiodistal and vertical directions (37).
- The number of lost teeth in both dental arches was recorded (the absence of third molars was not considered a loss of teeth) (38). Subjects were grouped according to the number of teeth lost: ≤4 and ≥5 (16).

### Assessment of periodontitis stage and grade

Stage and grade of periodontitis were assessed by an experienced periodontist (NB) based on the guidelines of the 2017 World Workshop on the Classification of Periodontal and Peri-implant Diseases and Conditions (15, 16). Stage III was recorded when: 1. interdental CAL ≥5 mm, 2. tooth loss due to periodontitis ≤4, 3. maximum PPD ≥6 mm, and 4. furcation involvement. Stage IV was recorded when additionally, to stage III, ≥5 teeth were lost due to periodontitis, masticatory dysfunction, secondary occlusal trauma, bite collapse, drifting, and flaring were observed, or less than 20 teeth remained. Periodontitis grade, describing the rate of progression, was adjusted for smoking, diabetes, and age. Grade A (slow rate) was recorded for non-smokers and non-diabetics. Grade B (moderate rate) when smoking <10 cigarettes/day and/or controlled diabetes (blood sugar levels as low as normal using medication); Grade C (rapid rate) when ≥10 cigarettes/day. Subjects with uncontrolled diabetes were not included in the study. Grade C was also recorded for early onset of the periodontitis assessed by bone loss/age ratio (15, 16).

### Orthodontic examination

#### Primary malocclusion—evaluation in maximum intercuspation

Primary malocclusion was assessed on all three planes: sagittal, vertical, and transversal.

Sagittal plane

1. Sagittal malocclusion was diagnosed according to the canine relationship, because 50.8% of first molars on the right and 45.8% on the left were lost in the subjects of the study sample. The threshold of 2 mm of canine displacement from Class I was used to define Classes II and III (39, 40).
2. The sagittal incisor relationship was evaluated based on the relation of the mandibular incisor tip and cingulum plateau of the maxillary central incisors. Class I, Class II div 1, Class II div 2, and Class III were defined (39).
3. Overjet (OJ) was measured assessing the horizontal incisors’ relationship (41).
  a) Normal OJ—1 to 4 mm;
  b) Increased OJ—≥5 mm;
  c) Decreased OJ—<1 mm.

Vertical plane

1. Overbite (OB)—vertical OB of incisors (41).
  a) Open bite—<0 mm;
  b) Normal OB—0–4 mm;
  c) Deep bite—≥5 mm.

Transversal plane
  If present, crossbite (anterior and posterior) was recorded (41).

Primary malocclusion was recorded as follows:

a) No malocclusion—canines in Class I relationship and no vertical, transversal or intra-arch anomalies were observed.
b) Class I malocclusion—canines in a Class I relationship, but vertical and/or transversal or intra-arch anomalies were observed.
c) Class II div 1 malocclusion—canines ≥2 mm Class II, and no retroclination of maxillary incisors was found.
d) Class II div 2 malocclusion—canines ≥2 mm Class II and Class II div 2 relationship of incisors was observed.
e) Class III was recorded—canines were ≥2 mm Class III and/or OJ ≤0 mm.
f) Subdivision Class II—an asymmetric canine relationship (Class II on one side and Class I on the other) was found (42).

#### Functional occlusal examination

Functional occlusion was assessed by evaluating lower jaw movements during protrusion and laterotrusion. For visualization of occlusal contacts, 8 µm foil was used (43).

a) Protrusion: incorrect guidance was registered if only a single incisor or other teeth than incisors guided. The acceptable/correct anterior guidance path was recorded if two, three, or all four incisors were in contact during lower jaw movement and all posterior teeth were disoccluded (44).
b) Laterotrusion: lateral movement was considered correct if only canines (canine guidance) or lateral teeth (posterior teeth group function) of the working side were in contact during the function. Incorrect lateral guidance was registered when incisors guided or contacts were present on the non-working side (44).

#### Assessment of occlusal trauma

Anterior teeth with secondary occlusal trauma were recorded when contacts between maxillary and mandibular incisors in maximum intercuspation were detected using 8 µm occlusal foil (43). During maximum intercuspation, anterior teeth either have light contact or are slightly out of contact according to the principles of mutually protected occlusion (44). One more test: fremitus (vibration of the tooth root) was assessed by manual palpation of the labial side of the anterior tooth during clenching to maximum intercuspation (45).

#### Evaluation of secondary malocclusion

PTM:

The diagnosis of PTM was based on the main complaints of the subject, the presence of occlusal trauma and/or spacing/flaring/extrusion between teeth (35).

1) Isolated spacing was recorded when spaces (≥1 mm) were observed in isolated interdental spaces, mesial or distal to a tooth or teeth that were exposed to occlusal trauma (34).
2) Diastema was recorded as a presence of developing spacing between two central incisors, which was not present in the past or existed but increased (38).
3) Flaring was recorded as multiple spaces between anterior teeth with protrusion caused by labial migration of the teeth (34).
4) Extrusion/overeruption was recorded as migration of a tooth in the incisal direction (34).

#### Assessment of oral habits

a) Tongue habit was assessed (by an orthodontist EZ) by tongue position at rest and swallow pattern and was registered when both infantile swallowing and inferior tongue position at rest was detected (38, 46).
b) Clenching was registered based on anamnesis and palpation of painful and tensed chewing muscles (47).
c) Bruxism was registered based on self-report, palpation of chewing muscles and the presence of severe tooth attrition and enamel cracks (48).

#### OT need

The need for OT and occlusal rehabilitation was determined by two experienced orthodontists (authors AS and EZ) and judged in two ways.

Firstly, Dental Health Component (DHC) of Index of Orthodontic Treatment Need (IOTN) was applied for all subjects (49). Very great/great (Grade 5 or 4), borderline (Grade 3), and little/no (Grade 2 or 1) need for OT were registered.

Secondarily, OT need was assessed by clinical assessment of the severity of secondary malocclusion based on occlusal trauma (which could not be treated by alternative methods such as selective grinding), severe PTM (flaring and/or extrusion of anterior teeth) and/or impaired function (15, 50).

#### Data preparation

Analyses were based on the patient as the unit of measure. Measurement analysis included mean CAL and the percentage of the affected sites by CAL ≥5 mm (≤30, >30%) (51). Patients were grouped by gender (male/female) and age. Age was assessed as a continuous variable. As the prevalence of severe forms of periodontitis has been described to reach its peak at the age 40 years, for this study, we transformed subjects’ age into two categories of age groups (≤40 and >40 years) (52).

Variables were grouped as dependent to be used in a multivariate binary logistic regression analysis model for PTM: sites with CAL ≥5 mm were grouped ≤30/>30%; periodontitis of anterior teeth yes/no based on CAL ≥5 mm; OJ as increased (≥5 mm) and other (<5 mm); and similarly, OB as deep (≥5 mm) and other (<5 mm); and posterior loss of occlusion yes/no. The number of lost teeth was grouped ≤4 and ≥5 (15).

### Statistical analysis

#### Sample size

Sample size was based on the 7.8% prevalence of severe periodontitis (13). To be able to describe this group of adult patients with stage III–IV periodontitis, we aimed to collect 120 patients. The alpha level was set at 5%.

#### Statistical methods

Statistical analysis was carried out using SPSS software version 27.0 (SPSS Inc, Chicago, Illinois, USA). A *P* value less than 0.05 was considered significant.

Descriptive statistics were used to describe the characteristics of the study sample. Normality of every data set was checked using a Kolmogorov–Smirnov (BOM) test. When the distribution of variables was normal, a Student’s *t*-test was used to compare the quantitative sizes of the two independent samples. The Mann–Whitney *U* and Kruskal–Wallis tests were used to compare non-normally distributed variables. The chi-square test was performed to specify whether the relationship between two categorical variables was statistically significant. The interdependence of qualitative evidence was evaluated by chi-square criteria. Depending on the sample size, exact (for small size) and asymptomatic criteria were used.

Binary associations between variables were evaluated with a non-parametric Spearman or Kendall correlation coefficient. The odds ratio was determined by performing logistic regression.

The Cohen’s kappa coefficient and intraclass correlation coefficient (ICC) were used as a measure of reliability (53).

#### Reliability of measurements

The clinical attachment level (CAL) was selected for reliability measurements as recommended in the literature on the severity and prevalence of periodontitis (54). The calibration of the principal investigator (EZ) involved a total of 120 repeated measurements performed on 30 teeth, which were performed twice by an orthodontist (EZ) and periodontist (NB) and then compared with each other. The ICC yielded a value of 0.93 [95% confidence interval (CI): 0.87, 0.95: *P* = 0.0001], and interclass agreement between authors EZ and NB was 0.95 (95% CI: 0.92, 0.96; *P* = 0.0001).

Periodontist (author NB) calibrated with two other experienced periodontists for assessment of stage and grade. Inter-examiner reliability yielded Cohen’s kappa coefficient of value 0.92 and intra-examiner reliability of 0.97.

Calibration for assessment of PTM was performed by two experienced orthodontists (authors EZ and RL), which resulted in the values of Cohen’s kappa coefficient of 0.81 (for evaluation in maxillary anterior teeth) and 0.87 (for evaluation in mandibular anterior teeth).

Calibration for assessment of OTN according to DHC-IOTN was performed by two experienced orthodontists (authors EZ and AS). Inter-examiner reliability yielded Cohen’s kappa coefficient of value 0.88 and intra-examiner reliability of 0.95.

Any disagreement between the examiners was solved by thorough discussion.

## Results

### Participant flow

**Figure 1.**
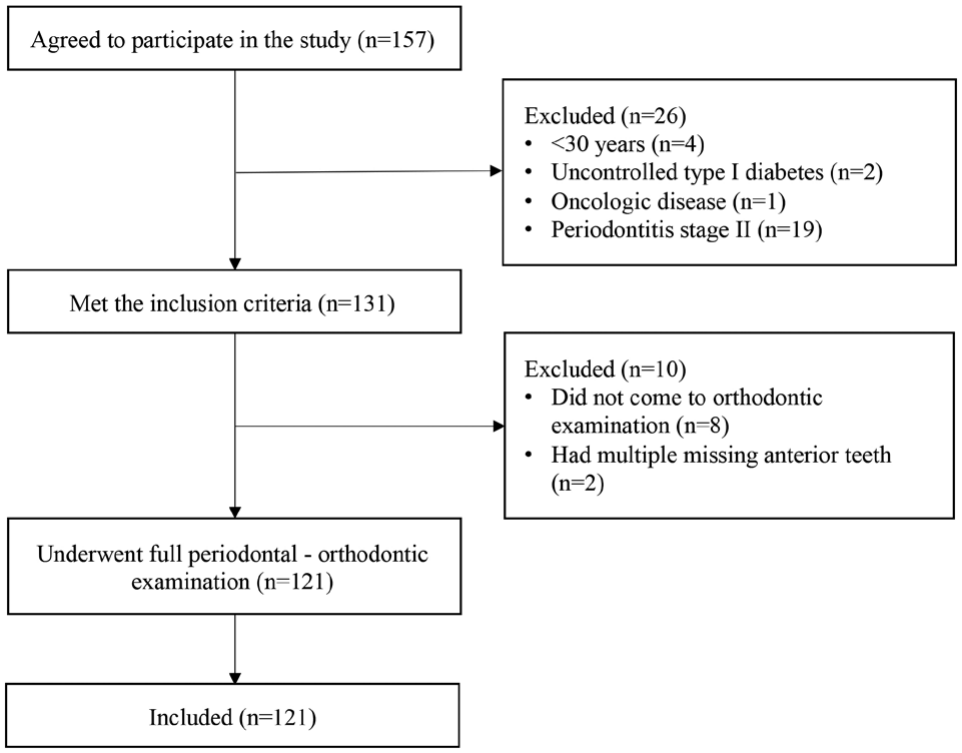
Flowchart of included subjects.

The selection period lasted between August 2020 and January 2022. One hundred and fifty-seven subjects agreed to participate in the study, 131 met the inclusion criteria, but 10 of these needed to be excluded. The flowchart of included patients is presented in Figure 1.

### Baseline data

A total of 121 subjects met the inclusion criteria and participated in the study, 70.2% females and 29.8% males, with a mean age of 46.2 years (95% CI: 44.4; 48.0, range 30–78), and 65.3% of subjects were older than 40 years. Only 62.8% of the subjects were systemically healthy. Cardiovascular diseases were the most prevalent conditions among those with systemic diseases. Systemic diseases mainly (82.2%) were reported in subjects >40 years (*P* = 0.002), and 20.6% of the subjects were smokers. Full baseline data can be seen in Table 1.

**Table 1.** Baseline data of the subjects enrolled in the study.

Variable	*n* (%)
Total	121 (100)
Gender	
Male	36 (29.8)
Female	85 (70.2)
Age (years)	
Mean (95% CI)	46.2 (44.4; 48.0)
Range	30–78
Systemic disease	
Endocrine/autoimmune disease	8 (6.6)
Cardiovascular disease	26 (21.5)
Other systemic disease	11 (9.1)
Systemically healthy	76 (62.8)
Smoking status	
Smoker ≥10 cigarettes/day	12 (9.9)
Smoker <10 cigarettes/day	13 (10.7)
Non-smoker	96 (79.4)
Previous orthodontic treatment	
Yes	18 (14.9)
No	103 (85.1)
Family anamnesis of periodontitis	
Yes	78 (64.5)
No	43 (35.5)
Clinical attachment loss (CAL) ≥5 mm	
≤30% sites	84 (69.4)
>30% sites	37 (30.6)
Anterior tooth mobility	
Yes	87 (71.9)
No	34 (28.1)
Periodontitis stage	
III	75 (62.0)
IV	46 (38.0)
Periodontitis grade	
A (slow rate)	48 (39.7)
B (moderate rate)	21 (17.3)
C (rapid rate)	52 (43.0)

### Results of the periodontal examination

Based on the case definition, the majority of subjects (62.0%), who participated in the study, were diagnosed with stage III periodontitis. An objective clinical examination yielded a mean CAL of 3.5 mm (95% CI: 3.31; 3.70) of included subjects, and 30.6 % of subjects had >30% of sites with CAL ≥5 mm (Table 1). Males were significantly more often found to have >30% of sites with CAL ≥5 mm than females, with an odds ratio of 2.9 [CI: 1.28; 6.63; *P* = 0.01].

Seventy-six per cent of subjects had at least one maxillary and 66.9% had at least one mandibular anterior teeth with PPD ≥6 mm and/or interdental CAL ≥5 mm. Age had no impact on the severity of periodontitis defined by stage (III or IV) (*P* = 0.11). However, subjects with age ≤40 years were significantly more (88.1%) likely to have Grade C (rapid progression) periodontitis (*P* = 0.02). On average, subjects had 2.7 teeth (CI: 2.21; 3.11) lost due to periodontitis. Unilateral posterior tooth loss was observed in 19.8% and bilateral tooth loss in 50.4% of subjects. Subjects >40 years had more than twice as frequent posterior tooth loss (72.0%) compared with subjects ≤40 (28.0%) (*P* = 0.02).

### Evaluation of complaints and malocclusions

The chief complaint of included subjects, who agreed to orthodontic examination, was the deterioration of smile aesthetics (Table 2).

**Figure 2.**
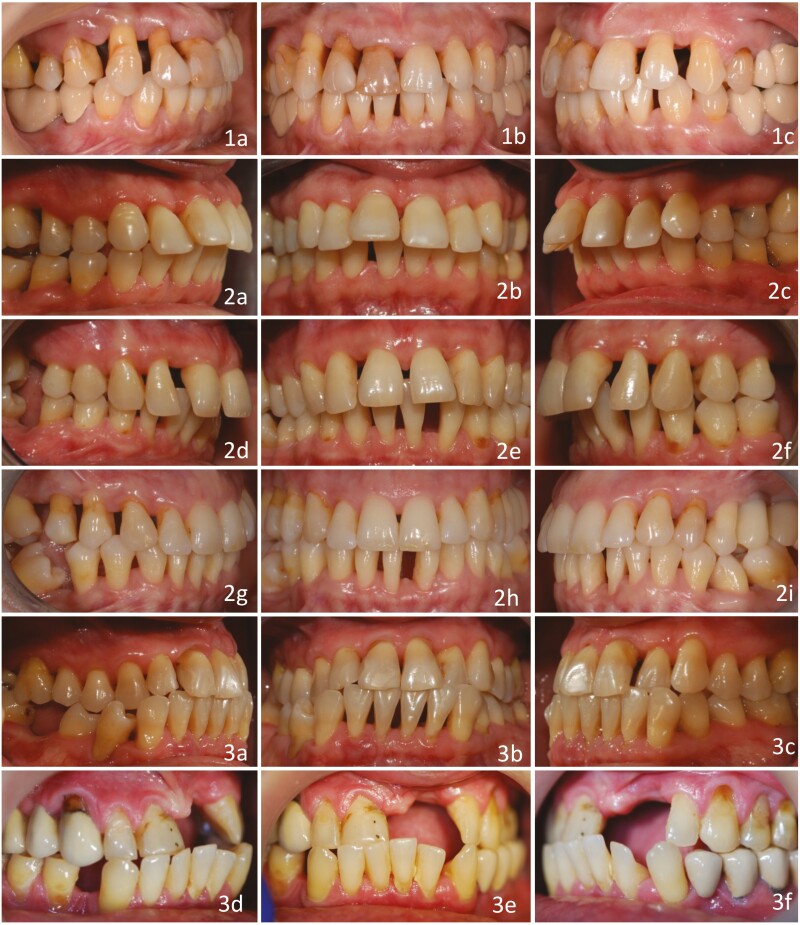
Examples of malocclusions in stage III–IV periodontitis. Class I: 1a–c; Class II div 1 with normal overbite (OB): 2a–c; Class II div 1 with severe occlusal trauma: 2d–f; subdivision Class II (asymmetric canine relationship): 2g–i; Class III with no tongue habit: 3a–c; Class III with lost tooth 21, tongue habit and severe pathologic tooth migration (PTM): 3d–f.

Analysis of sagittal canine relationship yielded that nearly half (49.6%) of the subjects had Class II malocclusion. However, a large part of them (45.0%) had subdivision II (Class II on one side and Class I on the other). 10.7% of subjects had a canine Class III relationship. Evaluation of sagittal incisor relationship resulted in a Class II div 1 incisor relationship in 22.3% of cases. Interestingly, 63.0% of cases with subdivision II of the canines had a Class I incisor relationship. Examples of malocclusions can be seen in Figure 2.

After adjusting all parameters for primary malocclusion evaluation, Class II div 1 malocclusion was found in 20.7% of subjects. Data on malocclusions can be seen in Table 2.

**Table 2.** Results of the orthodontic examination.

Variable	*n* (%)
Complaints	
Aesthetics	44 (36.4)
Function (and aesthetics)	18 (14.9)
Referred by the dentist	31 (25.6)
Other/many complaints	28 (23.1)
Canine classification	
Class I	48 (39.7)
Class II	33 (27.3)
Class II subdivision	27 (22.3)
Class III	13 (10.7)
Incisor classification	
Class I	68 (56.2)
Class II-1	27 (22.3)
Class II-2	12 (9.9)
Class III	14 (11.6)
Overjet	
<1 mm	12 (9.9)
1–4 mm	80 (66.1)
≥5 mm	29 (24.0)
Overbite	
<0 mm	3 (2.5)
0–4 mm	85 (70.2)
≥5 mm	33 (27.3)
Crossbite	
Anterior	11 (9.1)
Posterior	36 (29.8)
Anterior and posterior	5 (4.1)
Scissorsbite	1 (0.8)
Crowding (maxillary)	
Yes	39 (32.2)
No	82 (67.8)
Crowding (mandibular)	
Yes	66 (54.5)
No	55 (45.5)
Primary malocclusion	
Class I	38 (31.4)
Class II-1	25 (20.7)
Class II-2	12 (9.9)
Class II subdivision	23 (19.0)
Class III	13 (10.7)
No primary malocclusion	10 (8.3)
Anterior guidance	
Incorrect	99 (81.8)
Lateral guidance (right)	
Incorrect	103 (85.1)
Lateral guidance (left)	
Incorrect	97 (80.2)

Increased OJ (≥5 mm) was found in 24.0% of subjects; deep bite (≥5 mm) was found in 27.3% (*n* = 33) of subjects. When adjusted for a sagittal canine relationship, increased OJ was found only in 8.3% of Class II div 2 subjects and in 80% of Class II div 1 subjects; deep bite was found in 83.3% of Class II div 2 and 40.0% of Class II div 1 subjects (*P* = 0.001); open bite was found in only 3 subjects.

Posterior crossbite was found in 29.8% of subjects and was most prevalent in Class II malocclusion (61.1%), followed by Class I (30.6%) and Class III (8.3%).

Proclination of maxillary anterior teeth was observed in 48.8% (*n* = 59) of cases. Of them, 49.1% were found in Class II malocclusion (27.1% in Class II div 1 and 22.0% in subdivision II). Of all who had proclination (*n* = 59), 33.9% (*n* = 20) had lingual gingival recession (GR) on their maxillary central incisors, which was mostly observed together with Class II div 1 malocclusion (77.8%) (*P* = 0.001).

The percentage of subjects with no primary malocclusion was only 8.3%. Data on malocclusions can be seen in Table 2.

Sagittal canine malocclusion had no impact on the mean CAL either on all teeth (*P* = 0.63) or maxillary (*P* = 0.09) or mandibular anterior teeth (*P* = 0.69). No correlation between >30% or ≤30% of sites affected by CAL ≥5 mm and primary malocclusion was found (*P* > 0.05). Increased OB (*P* = 0.04) and OJ (*P* = 0.04) impacted higher mean CAL loss of maxillary anterior teeth.

### Results of functional occlusal examination

More than 80% of subjects had incorrect anterior or lateral guidance (Table 2).

### Occlusal trauma

At least one pair of anterior teeth with occlusal trauma was found in 82.6% of study subjects. There was no difference between occlusal trauma prevalence in stage III and IV periodontitis patients—81.3 and 84.8%, respectively (*P* = 0.63). 81.5 and 84.0% of subjects with periodontitis-affected maxillary and mandibular anterior teeth, respectively, had at least one pair of anterior teeth exposed to occlusal trauma. Occlusal trauma of anterior teeth was not influenced by sagittal malocclusion (*P* = 0.10), OJ (*P* = 0.62), OB (*P* = 0.46), or loss of posterior support of occlusion (*P* = 0.44).

### Secondary malocclusions—PTM

PTM was observed in 74.4% of maxillary and 60.3% of mandibular anterior dentitions and was not affected by age (≤40/>40 years) (*P* = 0.33 and *P* = 0.36, respectively), presence/absence of systemic disease (*P* = 0.51 and *P* = 0.74, respectively), or smoking/non-smoking (*P* = 0.22 and *P* = 0.67). However, PTM of mandibular teeth was found more in men (75.0%) compared with women (54.1%) (*P* = 0.03).

Two major types of PTM of anterior teeth were recorded—first, more prevalent was spacing, which was found in 56.2% (*n* = 68) of maxillary and 31.4% (*n* = 38) of mandibular anterior teeth within included subjects. Second, extrusion (40.5% in maxillary and 38.0% in mandibular) was found either with spacing, which was more prevalent in the maxillary anterior region (20.7% in maxillary versus 9.1% in mandibular) or with crowding, which was more prevalent in the mandibular anterior teeth (28.9% mandibular versus 19.8% maxillary). Interestingly, significantly more subjects with Class III malocclusion had spacing (84.6%), followed by Class I (63.2%) and Class II (46.7%), *P* = 0.03.

Overeruption of posterior teeth was found in 51.2% of subjects and mostly in cases of lost antagonist teeth.

### PTM in maxillary anterior teeth

The mean CAL of maxillary anterior teeth subjects with PTM was significantly greater (3.7 mm) compared with the mean CAL (3.1 mm) of anterior teeth in subjects without PTM (*P* = 0.04). PTM of maxillary anterior teeth was significantly more often observed in subjects with periodontally affected anterior teeth with CAL ≥5 mm (*P* = 0.001). Odds ratio for PTM of maxillary anterior teeth was 9.3 [CI: 2.07; 41.12; *P* = 0.001] in cases of >30% sites with CAL ≥5 mm.

In total, pathological maxillary diastema was observed in 29.7% of the subjects (developing maxillary diastema in 14.0% and diastema had always been present but has been increasing in 15.7%). Of those with periodontitis-affected maxillary anterior teeth (*n* = 92), 64.1% had spacing (31.5% had isolated interdental spacing and 32.6% flaring). A comparison of secondary malocclusions in periodontitis affected and periodontally healthy anterior teeth is available in Table 3.

**Table 3.** Comparison of secondary malocclusions in periodontitis affected and periodontally healthy maxillary and mandibular anterior teeth.

Secondary malocclusion	Maxillary anterior teeth			Mandibular anterior teeth		
	Periodontitis affected	Periodontally healthy	*P*	Periodontitis affected	Periodontally healthy	*P*
	*n* (%)	*n* (%)		*n* (%)	*n* (%)	
Spacing total	59 (86.8)	9 (13.2)	0.001	30 (78.9)	8 (21.1)	0.01
Minor	29 (76.3)	9 (23.7)		15 (65.2)	8 (34.8)	
Flaring	30 (100)	0 (0)		15 (100)	0 (0)	
No spacing	33 (62.3)	20 (37.7)		51 (61.4)	32 (38.6)	
Extrusion total	47 (95.9)	2 (4.1)	0.001	45 (97.8)	1 (2.2)	0.001
Extrusion with spacing	23 (92.0)	2 (8.0)		10 (90.9)	1 (9.1)	
Extrusion with crowding	24 (100)	0 (0)		35 (100)	0 (0)	
No extrusion	45 (62.5)	27 (37.5)		36 (48.0)	39 (52.0)	
Total subjects, *n*= 121	*n* = 92	*n* = 29		*n* = 81	*n* = 40	

Tooth extrusion was mostly registered in cases with periodontitis-affected teeth and occurred both in subjects with spacing and crowding (Table 3).

Crowding of maxillary anterior teeth was observed similarly in subjects with (31.5%) and without (34.5%) signs of periodontitis in the anterior region and was not related to periodontitis (*P* = 0.47).

### PTM in mandibular anterior teeth

The most prevalent type of PTM of anterior teeth in the mandibular dental arch was extrusion, observed in 38.0% of subjects. Notably, extrusion of mandibular anterior teeth more often appeared in periodontitis-affected crowded dental arches than in spaced ones (Table 3). However, there was no extrusion in crowded cases when teeth were periodontally healthy.

Spacing was not observed in 80.0% of subjects with periodontally healthy mandibular anterior teeth. On the contrary, if periodontally affected, 37.1% of subjects had spacing or flaring (*P* = 0.01).

Diastema in the mandibular anterior region was observed in 7.4% (*n* = 9) subjects.

Crowding of mandibular anterior teeth was observed similarly in subjects with (56.8%) and without (50.0%) signs of periodontitis in the anterior region and was not related to periodontitis (*P* = 0.31).

### Oral habits

Only 10.4% of subjects had bruxism, and 21.2% had clenching. However, tongue habit was mostly observed (43.2% of subjects). Tongue habit was significantly associated with spacing in the lower anterior region and was observed in 56.8% of subjects with periodontitis-affected mandibular anterior teeth compared with 19.5% with no tongue habit (*P* = 0.001).

### PTM in relation to factors

Factors influencing spacing of anterior teeth:

A bivariate logistic regression analysis yielded the following risk determinants significantly associated with spacing in maxillary anterior teeth: >30% of sites with CAL ≥5 mm (OR = 2.8), periodontitis-affected maxillary anterior teeth (OR = 4.0), number of lost teeth ≤4 (OR = 4.1) or ≥5 (OR = 6.9), posterior loss of occlusion (OR = 2.7), and Class III malocclusion (OR = 4.9). Risk determinants associated with spacing in mandibular anterior teeth included: number of lost teeth (OR = 3.5 if ≤4, and OR = 6.2 if ≥5 teeth lost), posterior loss of occlusion (OR = 3.9), Class III malocclusion (OR = 6.6), and tongue habit (OR = 5.7) (Table 4).

**Table 4.** Results of the bivariate logistic regression analysis for risk association of spacing in maxillary and mandibular anterior teeth.

Risk determinants	Spacing in maxillary anterior teeth		Spacing in mandibular anterior teeth	
	OR (95% CI)	*P*	OR (95% CI)	*P*
Clinical attachment loss (CAL) ≥5 mm				
≤30% sites	1.0		1.0	
>30% sites	2.8 (1.2; 6.6)	0.01	1.1 (0.5; 2.5)	0.87
Periodontitis in anterior teeth				
Periodontally healthy	1.0		1.0	
Periodontitis affected (CAL ≥5 mm)	4.0 (1.6; 9.7)	0.002	2.4 (1.0; 5.8)	0.58
Number of lost teeth				
No loss	1.0		1.0	
≤4 teeth	4.1 (1.6; 10.3)	0.003	3.5 (1.1; 11.3)	0.03
≥5 teeth	6.9 (2.1; 23.3)	0.002	6.2 (1.6; 23.4)	0.007
Posterior loss of occlusion				
No loss	1.0		1.0	
Unilateral/bilateral	2.7 (1.2; 6.1)	0.01	3.9 (1.4; 11.1)	0.007
Class III canine malocclusion				
Class I/Class II	1.0		1.0	
Class III	4.9 (1.0; 23.1)	0.04	6.6 (1.9; 23.2)	0.003
Tongue habit				
No	1.0		1.0	
Yes	1.5 (0.7; 3.1)	0.30	5.7 (2.5; 13.2)	0.001

Spacing in maxillary anterior teeth was not associated with tongue habit (Table 4), increased OJ (*P* = 0.90) and deep OB (*P* = 0.82), incorrect anterior guidance (*P* = 0.86), incorrect lateral (right and left) guidance (*P* = 0.65 and *P* = 0.82, respectively), and occlusal trauma (*P* = 0.92). In mandibular anterior teeth, no association was found between spacing and increased OJ (*P* = 0.34), deep OB (*P* = 0.55), incorrect anterior guidance (*P* = 0.64), incorrect lateral (right and left) guidance (*P* = 0.85 and *P* = 0.82, respectively), and occlusal trauma (*P* = 0.83).

Analysis showed multicollinearity between the loss of posterior support of the occlusion and number of lost teeth (*r* = 0.9, *P* = 0.001); therefore, we decided to test the number of lost teeth (≤4/≥5). Finally, sagittal malocclusion, periodontitis of anterior teeth and the number of lost teeth were tested.

Multivariate logistic regression yielded (overall percentage of the model, 68.6%) a higher odds ratio for spacing of maxillary anterior teeth, if they were periodontitis affected [OR 3.3; 95% CI: 1.29; 8.37, *P* = 0.01], had missing teeth [if ≤4 teeth missing—OR 3.6; 95% CI: 1.41; 9.39, *P* = 0.008 and OR 5.7; 95% CI: 1.64; 19.68, *P* = 0.006 if missing ≥5 teeth] and Class III malocclusion [OR 5.7; CI: 1.1; 28.8, *P* = 0.03].

For spacing of mandibular anterior teeth, a logistic regression model included sagittal malocclusion of canines and tongue habit. Multivariate logistic regression yielded (overall percentage of the model, 73.9%) a higher odds ratio for spacing of mandibular anterior teeth in subjects with Class III malocclusion [OR 6.9; 95% CI: 1.77; 26.91, *P* = 0.005] and tongue habit [OR 5.05; 95% CI: 2.02; 12.63, *P* = 0.001].

Factors influencing extrusion of anterior teeth:

The same factors as for spacing were tested for extrusion of anterior teeth; however, the odds ratio for extrusion was found only if the anterior teeth were affected by periodontitis [OR 14.1; 95% CI: 3.2; 62.8; *P* = 0.03] and if >30% of sites were affected by CAL ≥5 mm [OR 2.6; 95% CI: 1.19; 5.81, *P* = 0.016].

A bivariate logistic regression analysis did not show previous OT as a risk determinant for PTM of maxillary (*P* = 0.35) or mandibular (*P* = 0.33) anterior teeth.

### OT need

According to DHC of IOTN, very great OT need (Grade 5) was judged for 9.1%, great (Grade 4) for 49.6% and moderate (Grade 3) for 21.5% of subjects. 76.9% of subjects with Class III, 68.3% with Class II, and 44.7% with Class I malocclusion were judged to have great or very great OT need (Grade 4 or 5) (*P* = 0.55).

When assessed for secondary malocclusions (PTM, severe occlusal trauma, and/or impaired function), as many as 66.1% (*n* = 80) of subjects were judged in need of OT (91.3% of stage IV and 50.7% of stage III). Sixty per cent (*n* = 48) of them agreed to undergo OT. When OT need due to secondary malocclusions was analysed in sagittal malocclusions, subjects with Class III malocclusion needed treatment the most (92.3%) compared with Class II (73.3%) and Class I (55.3%) (*P* = 0.03).

Comparing both methods (DHC-IOTN and clinical assessment of secondary malocclusions), all subjects of DHC-IOTN Grade 5, 80.0% of subjects of Grade 4 and 53.8% of Grade 3 also needed OT due to secondary malocclusion. Subjects with Class II or III malocclusions needed orthodontic correction significantly more often, based on both assessment methods, DHC-IOTN [OR 3.3; CI: 1.20; 9.14, *P* = 0.02] and secondary malocclusions [OR 2.7; CI: 1.15; 6.17, *P* = 0.02] compared with subjects with Class I malocclusion.

## Limitations

Despite the relatively high number of subjects who agreed to participate in the study during the study period, 23% (*n* = 36) of them needed to be excluded (Figure 1). We aimed to collect special data on subjects ≥30 years and advanced stages of periodontitis, which has been reported to be between 7.8 and 9.8% of the adult population (13, 14). Most of the other studies usually include younger subjects (cut-off age of 17–18 years), and all stages (I–IV) of periodontitis severity in order to increase the sample size (35, 55). The low prevalence of the disease and the Covid-19 pandemic could have influenced the number of included participants in the present study. We observed that subjects, who declined to participate during an initial consultation, were afraid to be infected with Covid-19 during the additional appointment, which was needed to perform a full periodontal–orthodontic evaluation. So, during the course of the study, it was decided to perform a full periodontal and orthodontic examination at the same time as the consultation. This decision increased the rate of positive response.

One of the limitations was that X-rays, due to ethical reasons, were not performed for all included subjects. Therefore, for some subjects, grading of periodontitis was performed based on other valid clinical parameters listed in the guidelines (16).

One more limitation was that Angle classification of malocclusion was not applicable due to the large percentage of lost first molars (due to periodontitis), and therefore, primary malocclusion was based on the sagittal relationship of canines and incisors.

## Discussion

The present study is the first cross-sectional study analysing orthodontic anomalies in all three planes of space in subjects with stage III–IV periodontitis. The research is in high demand due to vastly increasing interest in OT by both dentists and patients, as shown in the literature (26). However, there is a lack of data on malocclusion prevalence and also influencing factors. The present study included not only periodontal but also orthodontic evaluation, including assessment of primary and secondary malocclusions. Stage IV periodontitis, which was found in 38% of the included subjects, is a particularly severe advanced form of the disease that, if not treated, leads to several or multiple tooth losses, secondary occlusal trauma and drifting and flaring of teeth. More importantly, implants, which are often used to replace lost teeth, have been shown to have a high risk for the development of peri-implantitis in subjects with periodontitis (56). Therefore, timely periodontal treatment is essential for saving natural teeth. Malocclusions have been described as co-destructive factors on the faster progression of CAL (11, 12, 57).

In the present study, canine and incisor relationship was used for the final judgement of primary malocclusion as the majority of first molars on both sides had already been lost. The results yielded that Class II malocclusion was observed in 49.6% of subjects.

Class II div 1 was found in only 21% of included subjects, which is similar to the adolescent population; however, increased OJ was 80% and deep bite (OB) was only 40% of them (41). A number of subjects had subdivision Class II (asymmetric class of canines), where OJ was normal (63% had a Class I incisor relationship). Class II div 2 was found in 9.9% of subjects and mostly together with increased OB (≥5 mm). More importantly, Class III also had quite high prevalence (10.7%), which was higher than the prevalence found among younger population in Europe (6.2%) (58). This may be partly explained by the loss of posterior height of occlusion. Class III has also been associated in the literature with thin gingiva and increased risk for GR, especially in the lower anterior region (59). Furthermore, OT in periodontally healthy Class III subjects with GR did not influence their improvement (60). Knowing that subjects with stage III–IV periodontitis may already have attachment loss in the lower anterior region, one should keep in mind the risk for further deterioration of soft tissues as tooth movement increases the risk for labial alveolar bone loss and consequently GR (30). In contrast, the literature emphasizes Class II as a major risk for PTM, which could not be confirmed by the results of the present study (22, 61). However, it can be seen in the present study that Class III malocclusion had a high odds ratio for PTM, such as spacing and/or flaring, especially in subjects with tongue habit. As previously discussed, risk for PTM in Class III subjects has never been discussed in earlier literature.

At least one pair of anterior teeth with occlusal trauma, which was assessed in maximum intercuspation of anterior teeth in the present study, was found in more than 80% of subjects (44). Occlusal trauma was registered as a heavy contact of anterior teeth when the *fremitus* of the root was present during palpation (11, 62). It is well described in the literature that heavy occlusal contacts induce risk for further periodontal breakdown and migration of teeth, especially in cases of untreated periodontitis (12). The literature has also described that teeth, exposed to traumatic occlusal interferences, have worse healing after periodontal therapy (10).

Spacing was the most prevalent secondary malocclusion found in the present study, followed by extrusion. The results of the present study yielded that anterior teeth with periodontitis had more PTM than periodontally healthy anterior teeth. Furthermore, reduced clinical attachment levels were associated also with deep OB and increased OJ. This is in line with the earlier literature, which has also suggested that spacing, deep OB, and increased OJ in adult subjects might be related to deeper pockets and increased CAL (11, 63). Other studies also state that general spacing, flaring, and extrusion of single teeth is typically seen, especially with local periodontal disease (64). In the present study, the odds ratio for spacing in maxillary anterior teeth was associated with Class III malocclusion, loss of teeth, and periodontitis of anterior teeth.

It is described in the literature that the nature of tooth migration may vary in different malocclusions and may depend on soft tissue parafunctions (38). The present study found that the most prevalent oral habit was tongue habit (43%), which influenced spacing of the mandibular anterior teeth. In subjects with tongue habit, teeth may not only procline but may also end up in the open bite malocclusion. However, open bite in the present study was found in only three subjects.

Literature on the prevalence of various types of PTM is scarce. Martinez-Canut *et al.* only studied PTM cases with diastema, which either appeared or was increasing; however, in the present study, it can be seen that diastema was not so prevalent (38).

The literature states that periodontally healthy anterior teeth bear the entire load on excursive movements of the mandible. If posterior teeth are lost, the load on the anterior teeth increases even more. Due to the loss of attached tissues, the movements of the mandible become too heavy and traumatizing for anterior teeth with reduced periodontium (34, 44). In the present study, incorrect anterior and lateral guidance was very prevalent; however, its association with PTM was not found.

Pathological migration of teeth and impaired smile aesthetics were found to be the main reasons for subjects to seek OT (26). In the present study, OT need was firstly determined according to the DHC of IOTN, where more than half of the subjects were determined to be in a great or very great OT need. Additionally, in 21.5% of cases, OT need was moderate. However, in the process of applying DHC-IOTN on the present sample, we encountered some difficulties that did not reflect real need of orthodontic tooth movement due to secondary malocclusions. One of them was the assessment of the extrusion of a maxillary and/or mandibular anterior tooth with periodontitis resulting in severe secondary occlusal trauma (*fremitus* in maximum intercuspation), functional problems, permanent tooth mobility, and further periodontal breakdown. So, the amount of displacement did not reflect the severity of secondary malocclusion, which, if not treated, would pose a risk for tooth loss in the long term. Furthermore, many of the subjects in the present study did not have an increased or OB with gingival contact (which would be of great OT need) but still had severe secondary occlusal trauma. One more problem was found that the loss of posterior support of occlusion was not included in the tool. Also, the IOTN does not take into consideration CAL and root disclosure. Finally, the original DHC-IOTN describes that subjects needing collaborative care, including restorative, periodontal, and orthodontic treatment, fall directly into Grade 4 (great OT need). So, application of other features would become unimportant (49)

Due to the previously listed reasons, final OT need in the present study was assessed not only by DHC-IOTN, but supplemented by clinical assessment of pathological changes due to the periodontal disease. It was assessed that 66.1% of subjects, due to secondary malocclusions, would benefit from OT. Interestingly, among them, 60% of subjects agreed to start OT, showing that consent to undergo OT in older ages is not a problem. However, one recent study found that possibilities of such treatment are not always presented to the patients (26). Extraction of multiple teeth may be considered as an alternative; however, the prevalence of peri-implantitis, especially in subjects with periodontitis, is bringing back the idea of saving natural teeth (31–33). OT in subjects with severe PTM is sometimes the only option (24).

Studies with larger sample sizes are needed to increase validity and generalizability of the findings of the present study.

## Conclusions

The most prevalent malocclusion was Class II. Spacing and extrusion were prevalent types of PTM, which were mainly influenced by CAL and tooth loss due to periodontitis. Preventive measures for PTM should be applied in cases with periodontally affected anterior teeth, before tooth extractions, in subjects with Class III malocclusion and tongue habit.

Orthodontists should be prepared for increased treatment demand as OT need, based on the results of the present study, was determined for more than half of the subjects. However, there is a lack of validated tools for assessment of OT need in adult subjects with advanced stages of periodontitis and secondary malocclusions.

## Data Availability

The data are available from the corresponding author, upon reasonable request.
